# Evaluation of Sensory Quality for Taiwanese Specialty Teas with Cold Infusion Using CATA and Temporal CATA by Taiwanese Consumers

**DOI:** 10.3390/foods10102344

**Published:** 2021-10-01

**Authors:** Shih-Lun Liu, Yih-Mon Jaw, Li-Fei Wang, George Chao-Chi Chuang, Zhen-Yu Zhuang, Yuh-Shuen Chen, Bo-Kang Liou

**Affiliations:** 1 Department of Food Science and Technology, Hungkuang University, No. 1018, Sec. 6, Taiwan Boulevard, Shalu District, Taichung City 43330, Taiwan; lourence@hk.edu.tw; 2 Department of Chinese Culinary Arts, National Kaohsiung University of Hospitality and Tourism, No. 1, Songhe Rd., Xiaogang District, Kaohsiung City 81271, Taiwan; eamon@mail.nkuht.edu.tw (Y.-M.J.); lifeiso@mail.nkuht.edu.tw (L.-F.W.); 3 Department of Food Science, China University of Science and Technology, No. 245, Academia Rd., Sec. 3, Nangang District, Taipei City 115, Taiwan; george0321@cc.cust.edu.tw; 4 Department of Food Science and Technology, Central Taiwan University of Science and Technology, No. 666, Buzih Rd., Beitun District, Taichung City 40601, Taiwan; 0956xxxxx5@gmail.com

**Keywords:** cold infusion, consumers’ acceptance, CATA, TCATA, tea, degrees of fermentation

## Abstract

The market size of varied carbonated teas and bottled ready-to-drink tea products in Taiwan has surpassed that of traditional Taiwan tea with hot infusion. The consumption behavior of Taiwanese consumers for new and varied types of cold infusion tea products has also differed from that of traditional hot infusion ones. More kinds of Taiwan tea with different fermentation levels are gradually being used as raw materials for various cold infusion tea products. Therefore, to study consumers’ responses towards cold-brewed tea has become more important for the market of tea in Taiwan. This study recruited Taiwanese consumers to taste seven Taiwanese specialty tea infusions with various degrees of fermentation, and their opinions were gathered by questionnaires composed of check-all-that-apply (CATA), and temporal check-all-that-apply (TCATA) questions and hedonic scales. We found that both CATA and TCATA data agreed that the sensory features of unfermented and lightly semi-fermented tea infusions could be plainly distinguished from the ones of heavily semi-fermented and fully fermented teas based on correspondence analyses. Through CATA and TCATA, the sensory characteristics of the cold-brewed tea of various fermentation degrees could be clearly identified. The first-hand information of cold tea beverages analyzed through this study could be useful for the development of the market in Taiwan. The proper level of bitterness, astringency, fresh tea leaf flavor, and late sweetness were the essential qualities of cold infusions brewed from lightly fermented teas, which could be the best raw materials for production of cold tea beverages to satisfy as many consumers as possible.

## 1. Introduction

As a major drink all over the world, fresh tea leaves (*Camellia sinensis*) traditionally are processed into various dried loose teas that are then steeped with hot water higher than 80 °C to acquire the hot infusions sipped by consumers. In the 1980’s, many newly developed cold tea beverages, such as varied carbonated teas, bubble teas, iced milk teas, and bottled ready-to-drink (RTD) tea products, started to appear in the market. The global RTD tea market size was valued at $29.66 billion in 2019 and is anticipated to reach $38.96 billion by 2027 [1], and the dense shops of various on-site-prepared cold tea beverages in many cities also disclose their popularity [2]. Taiwan is one of the few and important parts of the world producing various types of fermented tea. The RTD tea market in Taiwan exceeds 25 billion New Taiwan Dollars (NTD) per year and is dominated by young consumers [3]. The highest market ratio of RTD tea is green-tea-related products, and other cold tea with different fermentation is also growing. In addition, Taiwanese consumers are increasingly choosing sugar-reduced and sugar-free cold tea beverages [4] and are paying attention to the source of tea due to numerous incidents of pesticide residue. The origin of tea has become a key competitive factor for purchasing.

At present, most of the cold tea beverage products, whether they originated from standard operating procedures (SOP) factories or on-site shops, are prepared by brewing or even cooking the loose teas in boiling water to achieve the highest extraction efficiency, and the tea extracts of high concentrations are then diluted with cold water or ice for the ultimate service. However, more and more modern studies have revealed the thermal instability of certain tea compositions that contribute to the bioactivities of teas [5]. For instance, Lin, et al. (2008) discovered that after lyophilized and prepared into the same concentration (5 mg/mL), the green tea extracts from cold brewing (4 °C/24 h) performed more effective scavenging on DPPH and hydroxyl radicals as well as more potent chelating on ferrous ions than the extracts of hot brewing (90 °C/20 min) [6]. Additionally, because of the different interdependence between brewing duration and brewing temperature on various tea compounds [7], the tea compositions may eventually exist in dissimilar ratios in the cold and hot-brewed tea infusions, and this may result in their distinct sensory qualities. Therefore, even if made from the same loose tea, sensory characteristics of the tea infusions resulting from different temperature/time extractions may not be simply standardized by adjusting their water content.

It has been proposed to produce tea beverages with superior health effects by cold extraction. However, for beverage products, their sensory acceptability by customers may be a more decisive factor for their durability in the market. Even though the superior organoleptic qualities of cold-brewed tea infusions have been noted in some previous reports [8,9], there is still not enough information, especially customers’ direct responses to the sensory properties of cold-brewed teas, for expanding the application of cold extraction to the tea business. With advanced processing techniques of fresh tea leaves and as the origin of many modern cold tea beverages (e.g., bubble tea), Taiwan has economically benefited from various tea products. To characterize the sensory properties of cold-brewed infusions of Taiwanese teas, seven Taiwanese specialty teas representing various fermentation degrees were used in this research. To gather first-hand opinions from consumers, the check-all-that-apply (CATA) method was used for sensory evaluations performed directly by untrained respondents [10]. In addition, temporal check-all-that-apply (TCATA), an extension method of CATA, was used to track the dynamic sensory changes of these cold tea infusions over time [11]. Additionally, hedonic test and preference ranking were coupled to compare the sensory pros and cons of these seven cold tea infusions to explore the fermentation degrees of loose teas suitable for production of cold infusions.

## 2. Materials and Methods

### 2.1. Tea Samples

Seven Taiwanese specialty teas were used, sorted in order from low to high fermentation degrees as Bi-Lo-Chung (BLC), Wenshen Paochong tea (WSP), High-mountain tea (HMT), Dong Ding Oolong tea (DDO), Tie-Guan-Yin (TGY), Baihao Oolong tea (BHO), and Sun-Moon-Lake black tea (SML). Briefly, BLC is unfermented green tea, WSP, HMT, and DDO are lightly semi-fermented oolong teas, TGY and BHO are heavily semi-fermented oolong teas (TGY is a semi-fermented and heavily roasted), and SML is fully-fermented black tea. The information for these tea samples is shown in Table 1. Their individual detailed information can be found in our previous report [12]. The loose teas of similar moderate level of quality were purchased and prepared into cold-brewed tea infusions for this research. For sensory evaluation, Tea Research and Extension Station (TRES) of Taiwan has standardized the preparation procedures for various tea infusions. To prepare cold tea infusions, TRES recommended to mix 50 times the volume of room-temperature water with tea leaves in 2.5 L jars, and let stand at 2–7 °C for 4–10 h (4 h for green teas, 6 h for WSP, DDO, and TGY, 8 h for HMT, and 10 h for BHO and black teas) [13]. To unify the experimental operation to avoid unexpected variables, all of the cold tea infusions for this research were soaked statically in the sealed jars at 4–5 °C for 6 h, which was determined by pre-assessment. After 6 h of brewing, the tea leaves were quickly removed with strainers, and the tea infusions were sealed and restored in the refrigerator until used. All of the infusions were freshly prepared one day before implementation of sensory evaluations.

### 2.2. Questionnaire Design

For static sensory evaluations in which the factor of perception time was not considered, 54 total descriptive terms regarding color, aroma, flavor, mouthfeel, and aftertaste of tea infusions were adopted, and they were presented to the respondents in the format of checkbox questions. Definitions of these 54 terms were selected according to the researchers’ consensus from the original 175 descriptive terms explained in our previous report [12]. Additionally, the 9-point hedonic scale was used to investigate participants’ acceptability of each tea infusion sample [14]. Afterwards, respondents were instructed to rank their preference for these seven tea infusions based on the on-site experience. In contrast to the preference ranking resulting from actual sample tasting, a consumer behavior survey was also incorporated to measure participants’ intention to purchase these tea products according to their ideas about Taiwanese specialty teas. Both preference and purchase intention rankings were expressed from 1 to 7. Lower scores represented higher preference or purchase intention.

Another evaluation was also performed to explore the dynamic sensory properties of cold tea infusions. To understand how these cold-brewed tea infusions are perceived in-mouth during consumption, the temporal check-all-that-apply (TCATA) method introduced by Castura et al. (2016) [11] was modified to investigate the evolution of in-mouth sensations. According to the Temporal Dominance of Sensation (TDS) methodology recommended by Pineau et al. (2012) [15], no more than 10 attributes should be simultaneously enclosed in a temporal test. Therefore, an attribute list composed of seven flavor (sweetness, sourness, bitterness, astringency, roasted, fresh tea leaf, and floral) and two mouthfeel (late sweetness and pungency) descriptive terms was designed. From the previous studies of several tea research papers [16,17,18], it was revealed that these nine attributes were frequently adopted as the dominant in-mouth sensations of tea extracts that could be also readily identified by most untrained assessors [12]. Through the software Compusense Cloud (Compusense, Inc., Guelph, Ontario, Canada), the onset and extinction times for each attribute noted by the participants could be recorded.

### 2.3. Procedure of Sensory Evaluation

The static and dynamic sensory evaluations were conducted separately by different participants on different occasions.

#### 2.3.1. Static Sensory Evaluation

The 108 volunteer participants, consisting of 69 males and 39 females, were invited from Central Taiwan University of Science and Technology (CTUST), National Chung Hsing University, National Kaohsiung University of Hospitality and Tourism, and Taiwan Agricultural Research Institute. Fifty of them were school/institute staff and the others were students. Regarding their tea drinking habits, 75% of the participants had tea at least once a month, whereas the others drank tea at a lower frequency (shown in Table 2). The habit of tea drinking has penetrated into all classes of people in Taiwan. In addition to traditional hot brewed tea, various types of newly developed cold tea beverages, such as carbonated teas, bubble teas, iced milk teas, and bottled RTD tea products are flourishing in the market in Taiwan. Almost all of the consumers had consumed cold brew tea in Taiwan (less than 5% in this study). The environment of the evaluation room was identical to our previous description [12]. All participants had to sign a consent form before the test began.

All experimental designs containing a questionnaire and sampling plan for the CATA test are described in the previous study [12]. Before the cold tea infusions were served to the respondents, they were removed from the refrigerator and allowed to reach ambient temperature. Before participants entered the evaluation environment, the seven infusion samples had been poured into 70 mL white porcelain cups marked by random three-digit numbers. They were presented to the respondents by a sequential monadic technique with William Latin square design. Because respondents were allowed to taste more than one sip for each sample in the static evaluation, greater amounts of tea infusions were supplied than for the dynamic evaluation.

Respondents were guided to assess the tea samples arrayed in front of them. Before tasting a new sample, participants had to clean their palates with crackers and pure water. To avoid confusion, the tea samples were evaluated individually, and the previous sample could not be re-tried after evaluation for the next sample was started.

For the CATA questions, the respondents only had to tick the attributes from the 54 pre-determined terms that they perceived applicable to the test samples; no consideration of intensity was required. After selection of CATA attributes for one sample was completed, the 9-point hedonic rating followed, of which scores 1 to 9 were defined in order as “dislike extremely” to “like extremely”, respectively [14]. The test procedures for different tea samples were not mixed. Whenever the tasting of one sample was completed, its porcelain cup was placed on the table at the position relevant to the previous samples in light of evaluators’ preference levels. After all the seven tea samples were tasted, their overall preference ranking could be acquired based on their array order on the table. After the static sensory evaluation was processed by the participants who were blind to the sample information, the evaluation questionnaires were withdrawn, and a consumer behavior survey was conducted to investigate their intention to purchase these seven Taiwanese specialty teas.

#### 2.3.2. Dynamic Sensory Evaluation

Sixty-three volunteer respondents, including 25 males and 38 females, were students of CTUST recruited by our campus posters. All participants had to sign a consent form before the test began. Ahead of the formal evaluation, the TCATA procedure was illustrated, and the respondents were guided to review the computer operation as well as the nine sensory attributes by practicing with one sample cup of tea. This practice was included not only to ensure participants’ proper comprehension but also to lower their psychological pressure. Afterwards, the participants were reminded to clean their palates with crackers and pure water before tasting a new sample, and then the seven tea samples in 30 mL white porcelain cups marked with random 3-digit numbers were served one by one to each participant by a sequential monadic technique of William Latin square design [19]. On the computer screen, the nine attributes were simultaneously presented in random order [11]. Respondents were asked to consume all of the infusion in one cup with one sip and naturally swallow it to begin the TCATA evaluation. Concurrently with the tea samples touching the evaluators’ mouths, the “Start” buttons were clicked, and the appearance and disappearance of these nine attributes were tracked within 30 s by rapidly selecting and deselecting the descriptive terms. Eventually, there were three possibilities for every attribute: (1) not selected (never perceived); (2) selected but later deselected (appeared but disappeared within 30 s); (3) selected and also not deselected (emerged and retained for longer than the evaluation period). Assisted by the software Compusense Cloud, selection and deselection of the attributes could be clearly discriminated by the word colors (orange and gray, respectively) displaying on the computer screen. For each sample, the computer record was set to stop automatically 30 s after the Start button was clicked.

### 2.4. Statistical Analysis

XLSTAT [20] was used for the following statistical analyses. Cochran’s Q test was used to compare the frequencies of attribute selection from the CATA questions, and post hoc analysis was conducted using multiple pairwise comparisons with the McNemar test (Bonferroni). Correspondence analysis with chi-squared distance was calculated on the attributes responded to by more than 20% of participants to measure similarities among the tea infusions. Consumers’ overall liking of the tea infusions averaged from the 9-point hedonic scores was analyzed by one-way ANOVA in conjunction with Tukey’s honestly significant difference (HSD) test. Agglomerative hierarchical clustering analysis with Ward’s method and automatic truncation based on entropy were applied to classify the respondents according to their homogeneous overall liking for tea consumption. The smoothed citation proportions curves, the charts of significant differences in citation proportions, and product trajectories were analyzed by TCATA data to understand the sensory properties of these seven cold-brewed tea infusions over time and significant differences between them.

## 3. Results and Discussion

### 3.1. Static Sensory Characteristics of the Seven Cold-Brewed Tea Infusions

**Figure 1:**
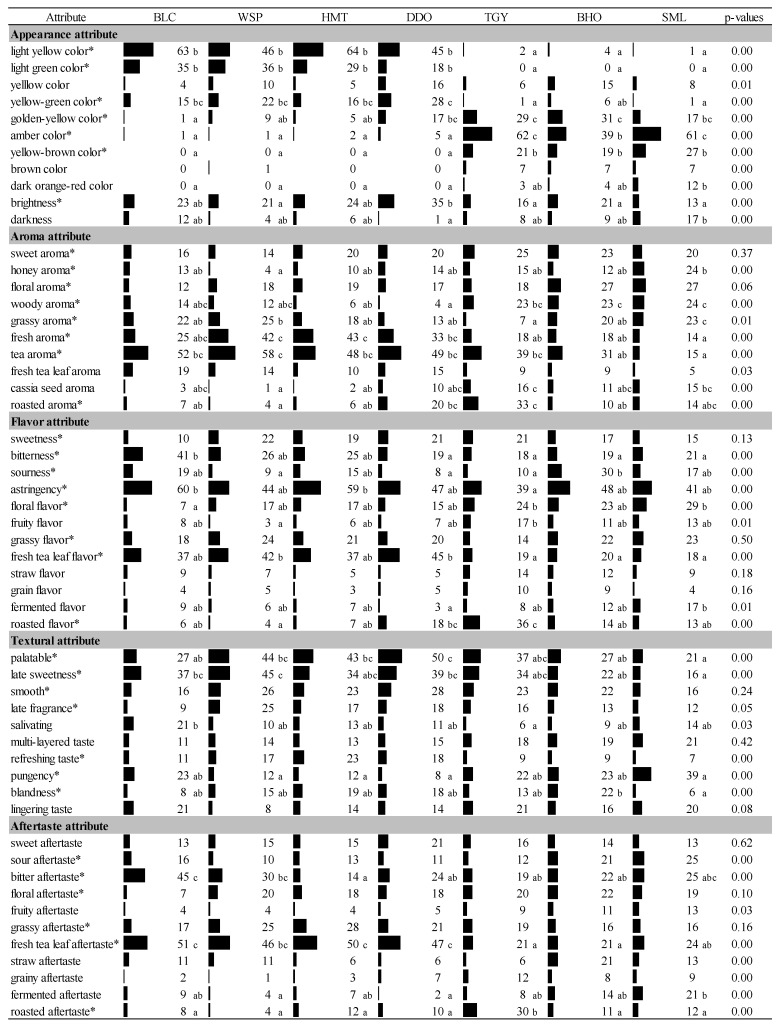
Selection frequencies of 54 sensory attributes for the cold-brewed infusions originated from seven Taiwanese specialty teas based on the check-all-that-apply (CATA) method (*n* = 108). BLC: Bi-Lo-Chung, WSP: Wenshen Paochong tea, HMT: High-mountain tea, DDO: Dong Ding Oolong tea; TGY: Tie-Guan-Yin, BHO: Baihao Oolong tea, SML: Sun-Moon-Lake black tea. Asterisks are marked on the attributes are significant at *p* < 0.05.

As shown in Figure 1, static sensory properties of these seven cold-brewed tea infusions were identified by respondents’ selection frequencies of the 54 sensory attributes, 41 of which displayed significant difference (*p* < 0.05) among the samples of distinct fermentation degrees. Even though this sensory evaluation was performed by different respondents from those who participated in our previous research for hot-brewed infusions [12], the data of these two studies could be comparable since they were obtained using the identical loose tea samples and were evaluated by the number of respondents essential for obtaining stable sample/descriptor configurations (*n* = 108 for this study; *n* = 109 for previous study) [21]. Many sensory properties were shown to vary similarly among these seven tea infusions, whether they were cold- or hot- brewed. For example, the fermentation strength between DDO and TGY caused certain distinct oxidation reactions in tea leaves, which made the infusions of lightly semi-fermented WSP, HMT, and DDO possess the sensory qualities similar to those of unfermented BLC, whereas the infusions of heavily semi-fermented TGY and BHO display the sensory properties close to those of fully fermented SML.

However, solubilities of various tea compounds like catechins and amino acids [22] may vary with temperature. In general, selection frequencies of many cold tea attributes were lower than those of hot teas, in agreement with Lin’s report that cold brewing resulted in the tea infusions of less color and milder organoleptic properties [8]. Even though much longer steeping time (6 h) was applied to cold-brewed tea preparation (hot brewing was processed with boiling water for 5 min [12]), it could be inferred from the low selection frequencies of certain sensory attributes that a few compounds could not be efficiently extracted from tea leaves by cold water. This phenomenon was especially evident in the heavily fermented teas (TGY, BHO, and SML) whose bar chart structures of cold-brewed infusions look significantly smaller when compared to those of their hot-brewed samples [12]. It has been reported that theaflavins and thearubigins are the major components contributing to the color and taste of heavily fermented teas [22,23]. Theaflavins, which display “bright orange-red” tone (defined as “amber” in our CATA questionnaire), are the condensed dimers of flavan-3-ols produced by enzymatic oxidation. As oxidation degrees are enhanced, more catechins and their gallates would be combined to form the larger polymers, thearubigins, which exhibit dark orange-red ~ brown colors [24]. Eventually in the fully fermented black teas, theaflavins and thearubigins would reach 1–6% and 10–20% of the dry weight of solids, respectively [25]. By comparing the selection frequencies of appearance attributes between cold and hot-brewed infusions of heavily fermented teas, it could be speculated from the similar amber color that theaflavins can be well dissolved in either cold or hot water. However, the notably lower selection frequencies of dark orange-red and brown colors could reflect the low solubility of thearubigins in cold water.

**Figure 2:**
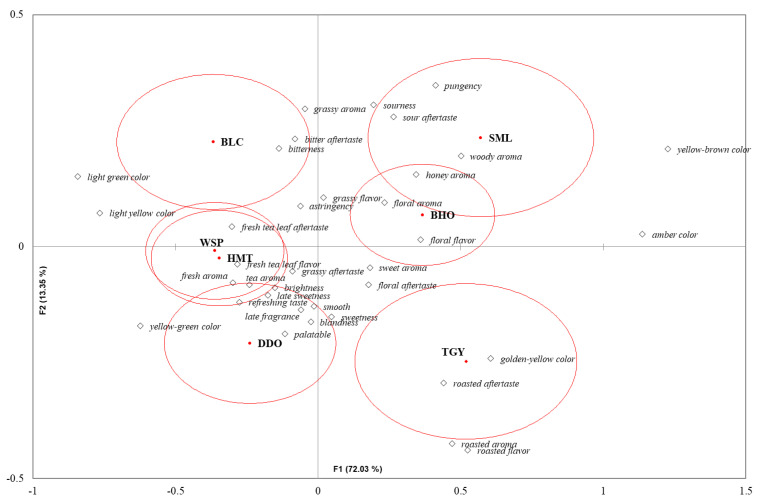
A bi-plot by correspondence analysis of the seven cold tea infusions in association with the 36 sensory attributes selected by more than 20% of participants. Significant difference (*p* < 0.05) of panelists’ selection proportions for each attribute among the seven tea samples were determined by Cochran’s Q test. The pairwise post-hoc McNemar test (Bonferroni) was also performed, and the tea samples signed with different alphabet letters were significantly different in the frequencies of panelists’ selection for the attribute listed in the same row. Asterisks are marked on the attributes selected by more than 20% of respondents.

According to Pareto principle, the attributes perceived by more than 20% of respondents could be recognized as the important characteristics, while the others were considered irrelevant [26,27]. The 36 terms marked with asterisks (*) in Figure 1 were the important attributes for cold-brewed tea infusions, and they were a little different from the 39 important attributes for hot teas determined by our previous research [12]. Besides dark orange-red color, brown color and darkness to be expelled from the appearance determinants, fresh tea leaf aroma (it’s different from “fresh tea leaf flavor” listed in the “flavor attribute” category of Figure 1), fermented flavor, multi-layered taste, lingering taste, and sweet aftertaste were also less perceivable in the cold infusions. On the contrary, yellow-green and golden-yellow colors in the cold infusions became more apparent, which could result from the better conservation of certain colored flavonoids, such as quercetin, under cold temperature [28]. In addition, sweet aroma, sourness flavor and sour aftertaste also became more influential organoleptic characteristics in cold-brewed teas. It may be interesting to explore the reasons by further analyzing and comparing the impactive chemical compounds in cold and hot-brewed tea infusions. Anyway, correspondence analysis of these 36 judgmental attributes for cold infusions with Chi-square distance calculation places these seven teas on the similar positions to the graph resulting from the hot-brewed samples (Figure 2) [12]. That is, green tea (BLC) and lightly semi-fermented oolong teas (WSP, HMT, and DDO) could be clearly divided from heavily semi-fermented teas (TGY and BHO) and fully fermented black tea (SML) according to the Factor 1 (72.03%). However different from that of hot-brewed samples, the cold-brewed BHO was more similar to SML according to the Factor 2 (13.39%). Much lower solubility of pungency compounds in cold water could be one of the elements which reduced the distance between BHO and SML. Disappearance of fermented flavor through roasted procedure could also lower the connection between BHO and TGY.

### 3.2. Hedonic Degrees for Individual Cold-Brewed Tea Samples

**Figure 3:**
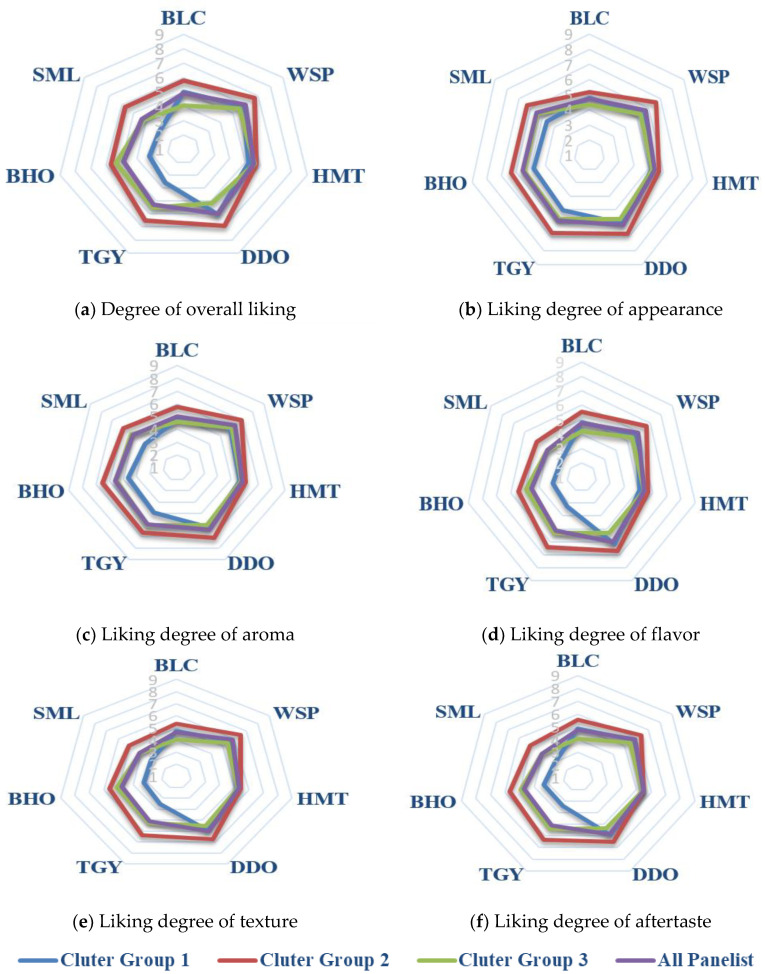
Preference degrees for the seven cold tea infusions revealed by a 9-point hedonic test in view of overall liking and individual sensory qualities. (**a**) Degree of overall liking; (**b**) Liking degree of appearance; (**c**) Liking degree of aroma; (**d**) Liking degree of flavor; (**e**) Liking degree of texture; (**f**) Liking degree of aftertaste.

The results of 9-point hedonic tests individually performed for each cold tea infusion were expressed by the 9-circle radar graphs of Figure 3. The nine circles from inner to outer represent scores 1 to 9, respectively. Through hierarchical clustering analysis of hedonic data, the respondents were classified into clusters 1–3, which accounted for 31.5%, 40.7%, and 27.8% of total participants, respectively. In view of “overall liking” (Figure 3a), all of the cold teas were scored between 5 (neither like nor dislike) and 6 (like slightly) by the people of Cluster 1 (red, 31.5%), and the cold infusions made of DDO, WSP, and TGY were their highest preference (*p* < 0.05). The consumers of Cluster 1 showed high acceptability for all tea samples, and they may be considered as the style of “drinking as long as it is tea”. The respondents of Cluster 2 (green, 40.7%) rated the seven cold tea infusions between score 4 (dislike slightly) and score 6 (like slightly), and they significantly preferred semi-fermented WSP, HMT, DDO, TGY, and BHO over fully fermented SML and unfermented BLC (*p* < 0.05). Therefore, the consumers of Cluster 2 may be regarded as “enthusiasts for semi-fermented teas”. The people of Cluster 3 (blue, 27.8%) evaluated all cold teas between score 2 (dislike very much) and score 6 (like slightly), and their acceptability degrees for various teas varied significantly (*p* < 0.05). As shown by the blue circle of Figure 3a, Cluster 3 respondents preferred WSP, HMT and DDO to TGY and BLC, and almost could not accept the most heavily fermented teas, BHO and SML. They may be taken as “picky consumers” to whom the heavily fermented teas should not be served. Generally, lightly semi-fermented teas may be the best raw materials for production of cold tea beverages to satisfy as many consumers as possible. By comparing the graph similarity between overall liking (Figure 3a) and the individual sensory elements (Figure 3b,f), it was disclosed that flavor, mouthfeel, and aftertaste were more correlated than appearance and aroma to consumer preference for cold tea infusions.

### 3.3. Preference Ranking vs. Purchase Intention

Respondents were requested to rank their preference and purchase intention for the cold infusions of these seven Taiwanese specialty teas with numbers from 1 to 7 to express the ranking order from highest to lowest. Therefore, the lower number represented the higher preference ranking or purchase intention. In Table 3, consumers’ purchase intentions for these seven teas, originating from respondents’ association towards the tea product names, were presented by the sum of 108 respondents’ rankings. However, six participants could not correctly complete their work, so preference rankings were eventually expressed with the sum of 102 respondents’ data. Additionally, multivariate statistical analysis was conducted to give RV coefficients, which take values between 0 and 1 to reveal the similarity between two sets of variables [29]. Through practical tasting without messaging, it was revealed that respondents significantly preferred the cold-brewed infusions prepared from the lightly semi-fermented teas (DDO > WSP > HMT > TGY > BHO > BLC > SML). However, the RV coefficient of 0.081 indicated that there was significant dissimilarity between the varying trends of preference ranking and purchase intention. Even though the sensory characteristics of WSP were recognized by many participants as one of the most preferred (not only this research for cold-brewed infusions but also our previous study for hot-brewed infusions divulged that WSP was the most liked overall among the Taiwanese specialty teas [12]), the commodity image of WSP does not match the real perception it supplied to consumers. It is recommended that WSP producers should enhance communication with consumers to promote their product’s impression in consumers’ minds.

### 3.4. Dynamic Sensory Characteristics of Cold-Brewed Tea Infusions

#### 3.4.1. Dynamic Sensory Features of Cold Tea Infusions Expressed with TCATA Curves

**Figure 4:**
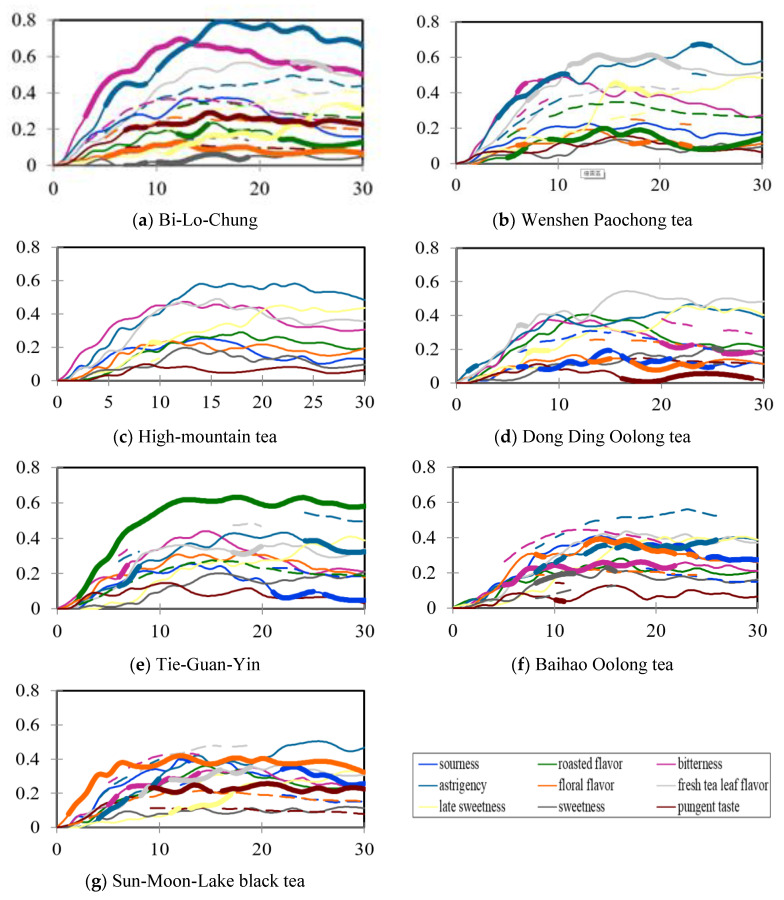
Smooth curves of temporal check-all-that-apply analysis of the nine selected sensory characteristics for the Taiwanese specialty tea infusions (*n* = 62). (**a**) Bi-Lo-Chung; (**b**) Wenshen Paochong tea; (**c**) High-mountain tea; (**d**) Dong Ding Oolong tea; (**e**) Tie-Guan-Yin; (**f**) Baihao Oolong tea; (**g**) Sun-Moon-Lake black tea. Dashed lines correspond, for the attribute of interest, to significant reference lines for all other Taiwanese specialty teas pooled together. Significant reference lines are displayed with a thicker line.

Dynamic sensory characteristics of the seven cold tea infusions were graphed as smoothed TCATA curves based on respondents’ selection frequencies of the nine attributes within 30 s (Figure 4a‒g) [11]. Within certain time slices, thicker lines were marked to indicate the significant difference between the selection frequencies of specific sample attributes and the reference data averaged from the other six pooled samples. For example, as soon as the cold BLC infusion was sipped, people could perceive its bitterness which then quickly turned significantly stronger than the reference intensity after 5 s, and this strong bitterness was sustained until the end of the evaluation (Figure 4a). To simplify the sensory feature identifications of individual teas with more concise TCATA graphs, reference lines of the nine attributes are not labelled in Figure 4a,g. However, the homogenous TCATA graph of HMT in which no thicker line was visualized (Figure 4c) may be referred as the average dataset of these seven cold tea infusions. From these TCATA curves, it may be interpreted that unfermented BLC was significantly characterized for its strong bitterness, astringency, pungency, as well as weak late sweetness and floral flavor (Figure 4a). Lightly semi-fermented WSP was significantly strong for its astringency in the early stage and high for its fresh tea leaf flavor and late sweetness in the middle stage. Generally, the WSP infusion was especially low in roasted flavor (Figure 4b). Compared to the other infusions, the DDO sample was overall low in sourness, and also weak for floral flavor, pungency, and bitterness in the middle and late stages (Figure 4d). As described in our previous report [12], processing of Taiwanese TGY tea leaves is well known for its delicate roasting techniques, and apparent roasted flavor was also preserved in its cold infusion (Figure 4e). The most heavily fermented oolong tea BHO was lower in bitterness and astringency but higher in sourness and floral flavor than the non-fermented or lightly fermented teas (Figure 4f). The cold infusion of fully fermented SML was especially significant for its floral flavor, which could be easily perceived and maintained for the whole evaluation period. The pungent taste, which turned significant after 10 s, was also one of its important sensory characteristics (Figure 4g).

#### 3.4.2. Pairwise Product Differences in TCATA Profiles

**Figure 5:**
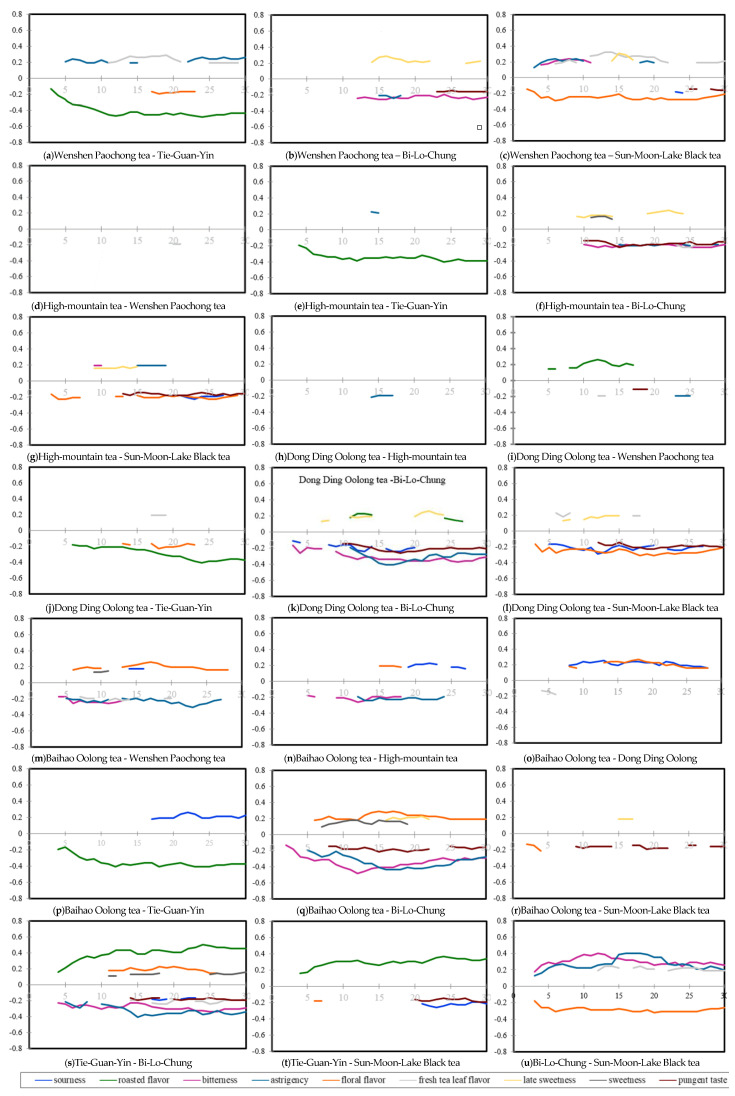
The significant discrimination in citation proportions of temporal check-all-that-apply curve for the nine selected attributes between Taiwanese specialty tea infusions are displayed for each product pair. (**a**) Wenshen Paochong tea–Tie-Guan-Yin; (**b**) Wenshen Paochong tea–Bi-Lo-Chung; (**c**) Wenshen Paochong tea–Sun-Moon-Lake Black tea; (**d**) High-mountain tea–Wenshen Paochong tea; (**e**) High-mountain tea–Tie-Guan-Yin; (**f**) High-mountain tea–Bi-Lo-Chung; (**g**) High-mountain tea–Sun-Moon-Lake Black tea; (**h**) Dong Ding Oolong tea–High-mountain tea; (**i**) Dong Ding Oolong tea–Wenshen Paochong tea; (**j**) Dong Ding Oolong tea–Tie-Guan-Yin; (**k**) Dong Ding Oolong tea–Bi-Lo-Chung; (**l**) Dong Ding Oolong tea–Sun-Moon-Lake Black tea; (**m**) Baihao Oolong tea–Wenshen Paochong tea; (**n**) Baihao Oolong tea–High-mountain tea; (**o**) Baihao Oolong tea–Dong Ding Oolong; (**p**) Baihao Oolong tea–Tie-Guan-Yin; (**q**) Baihao Oolong tea–Bi-Lo-Chung; (**r**) Baihao Oolong tea–Sun-Moon-Lake Black tea; (**s**) Tie-Guan-Yin–Bi-Lo-Chung; (**t**) Tie-Guan-Yin–Sun-Moon-Lake Black tea; (**u**) Bi-Lo-Chung–Sun-Moon-Lake Black tea.

Pairwise product differences are often investigated for product development. The TCATA data for each sample were also compared in pairs, and the differences in TCATA profiles of two products were obtained by taking their differences in TCATA selection frequencies for each of the attributes at every moment of the evaluation period [11] (Figure 5a–u). For examples, the three lightly semi-fermented oolong tea samples (WSP, HMT, and DDO), for which participants demonstrated similar levels of preference (Table 3), displayed little difference of TCATA characteristics from each other (Figure 5d,h,i). When WSP was compared to the heavily semi-fermented TGY (Figure 5a), TGY cold infusion showed stronger roasted flavor during the whole evaluation period, and its bitterness was especially obvious within 17–23 s, whereas WSP displayed stronger astringency in the early and late stages, and fresh tea leaf flavor was its important sensory feature in the middle stage. Compared to the unfermented BLC sample, late sweetness of WSP was a major characteristic in the second-half period, and almost simultaneously, BLC was significantly higher in bitterness, and its strong pungency appeared after 22 s (Figure 5b). In contrast to the fully fermented SML infusion, WSP tasted more bitter and astringent in the early stage, and more late sweetness was perceived in the middle and late stages, whereas SML was significantly recognized for its floral flavor for the whole evaluation period (Figure 5c). Significant floral flavor could be recognized in the heavily fermented tea samples (BHO and SML) when they were compared to the other unfermented or lighter fermented teas (Figure 5g,l,m,o,q,u). Even though floral flavor has often been emphasized as a positive aroma in the traditional hot tea business, our respondents’ evaluation revealed that floral flavor may not be indispensable for the quality of cold tea infusions, since BHO and SML were two of the samples ranked the lowest preference.

#### 3.4.3. Correspondence Analysis of TCATA Product Trajectories

**Figure 6:**
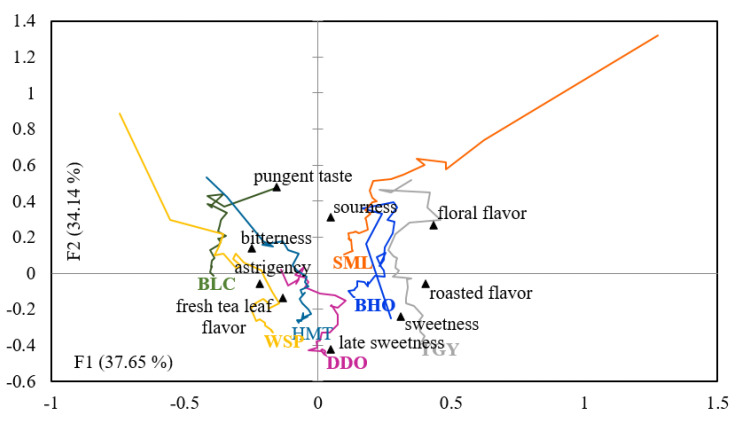
Trajectory CA graphical representations for Taiwanese tea beverages brewing with cold water on nine sensory attributes using temporal check-all-that-apply.

Correspondence analysis was also applied to track the TCATA trajectories of these seven cold tea infusions. As shown in Figure 6, factor 1 and factor 2, respectively, account for 37.65% and 34.14%, totaling 71.79% of the characteristic variables. All product trajectories moved over time and at the end were marked with the sample name. Similar to the result of the static CATA test (Figure 1), sensory trajectories of unfermented (BLC) and lightly semi-fermented (WSP, HMT, and DDO) tea infusions could be plainly distinguished from the ones of heavily semi-fermented (TGY and BHO) and fully fermented (SML) teas based on factor 1 (37.65%). Trajectories of the three lightly semi-fermented teas (WSP, HMT, and DDO) shifted in parallel with one another following the pattern of bitterness, astringency, fresh tea leaf flavor, and late sweetness. Coupled with participants’ preference ranking, it could be inferred that proper level of bitterness, astringency, fresh tea leaf flavor, and late sweetness are essential characteristics for sensory qualities of cold infusions brewed from lightly fermented teas. Even though unfermented BLC was perceived as having bitterness, astringency, and fresh tea leaf flavor, it might bring consumers a completely different perception due to extra pungency or deficient late sweetness. In contrast, floral flavor, roasted flavor, and sweetness were the sensory features located closer to the trajectories of three heavily fermented teas (TGY, BJO, and SML). It is unclear which attributes supply positive contributions to the sensory qualities of cold infusions of heavily fermented teas. However, these sensory elements did not seem to matter for the consumer preference once high intensity of sourness was produced by heavy fermentation. Among the three heavily fermented and roasted teas, fermentation degree of TGY was the lowest, and it was also the only one with the consumer preference level comparable to those of lightly semi-fermented teas (Table 3). Its distance from “sourness” might be one of the major reasons. Additionally, it was inferred from the especially intense roasted flavor perceived only in the TGY sample that “roasted flavor” may not be indispensable but could be advantageous for the sensory qualities of cold infusions brewed from heavily fermented teas.

## 4. Conclusions

Through CATA and TCATA, first-hand information for sensory characteristics of the cold-brewed teas of various fermentation degrees was collected from consumers. According to the Pareto principle, 36 important sensory attributes for cold-brewed tea infusions were selected by CATA test performed by 108 untrained respondents. After correspondence analysis of the 36 attributes and their selection frequencies, static sensory features of the seven cold tea samples were acquired. Compared to the result of our previous hot tea research in which tea samples were prepared by boiling water (5 min), it could be inferred that certain tea compounds (e.g., thearubigins) are not readily dissolved, whereas the others (e.g., quercetin) may be better preserved in cold water brewing (4–5 °C/6 h). The tea compositions of dissimilar ratios may result in the different sensory qualities of cold and hot-brewed tea infusions. Coupled with a 9-point hedonic test, it was disclosed that flavor, mouthfeel, and aftertaste were more important than appearance and aroma for consumer preference level of cold tea infusions. TCATA test performed by 63 respondents was also conducted to track the dynamic sensory changes of nine sensory attributes in cold tea infusions over time. Both CATA and TCATA data agreed that the sensory features of unfermented and lightly semi-fermented tea infusions could be plainly distinguished from the ones of heavily semi-fermented and fully fermented teas based on correspondence analyses. Even though TCATA allows simultaneous evaluation of no more than 10 attributes, it provided the detailed sensory fingerprint (trajectory) of each tea sample and its corresponding relationship with individual sensory attributes. Based on TCATA trajectories and preference ranking, it was revealed that proper levels of bitterness, astringency, fresh tea leaf flavor, and late sweetness were the essential characteristics composing the sensory qualities of cold infusions brewed from lightly fermented teas. Tea leaves of low fermentation degrees could be the best raw materials for production of cold tea beverages to satisfy as many Taiwanese consumers as possible. In contrast, floral flavor may not be a very influential element, but sourness is an obvious disadvantage for the sensory qualities of cold infusions brewed from heavily fermented teas. In addition, roasted flavor may not be indispensable but could be beneficial for those of heavily fermented teas.

## Figures and Tables

**Table 1:** The information for seven Taiwanese specialty teas in this study [12].

Full Name of Tea	Abbreviation	Classification	Fermentation Degree (%)	Origin
Bi-Lo-Chung	BLC	Green tea	0	New Taipei City
Wenshen Paochong tea	WSP	Oolong tea	12–15	New Taipei City
High-mountain tea	HMT	Oolong tea	20–30	Chiayi County
Dong Ding Oolong tea	DDO	Oolong tea	20–30	Nantou County
Tie-Guan-Yin	TGY	Oolong tea	30–40	Taipei City
Baihao Oolong tea	BHO	Oolong tea	60–70	Hsinchu County
Sun-Moon-Lake black tea	SML	Black tea	>90	Nantou County

**Table 2:** The characteristics of clusters of consumers in this study (*n* = 108).

Category		Group 1		Group 2		Group 3		Total
Sex	Female	22	64.71%	26	59.09%	21	70.00%	63.89%
	Male	12	35.29%	18	40.91%	9	30.00%	36.11%
Age	20–29	25	75.76%	36	83.72%	26	89.66%	81.90%
	30–39	5	15.15%	3	6.98%	2	6.90%	9.52%
	40–49	2	6.06%	3	6.98%	1	3.45%	5.71%
	50–59	1	3.03%	1	2.33%	0	0.00%	1.90%
Work	office worker	11	33.33%	12	27.91%	8	27.59%	29.52%
	housekeeper	1	3.03%	0	0.00%	0	0.00%	0.95%
	Student	21	63.64%	31	72.09%	21	72.41%	69.52%
Drinking tea habit	Not at all	2	6.06%	2	4.65%	1	3.45%	4.76%
	Not often	4	12.12%	9	20.93%	8	27.59%	20.00%
	Once per month	1	3.03%	3	6.98%	1	3.45%	4.76%
	Several time per month	3	9.09%	4	9.30%	4	13.79%	10.48%
	Once per week	2	6.06%	6	13.95%	5	17.24%	12.38%
	Several times per week	12	36.36%	15	34.88%	5	17.24%	30.48%
	Every day	9	27.27%	4	9.30%	5	17.24%	17.14%

**Table 3:** Preference ranking versus purchase intention for the cold infusions of these seven Taiwanese specialty teas.

Sample	Preference Ranking	Purchase Intention
BLC	^1^ 483 ^c^	441 ^b^
WSP	330 ^a^	472 ^b^
HMT	367 ^a^	349 ^a^
DDO	307 ^a^	388 ^ab^
TGY	385 ^ab^	401 ^ab^
BHO	464 ^bc^	403 ^ab^
SML	521 ^c^	403 ^ab^
RV coefficient	0.081	

^1^ Values in the same column with different superscripts letters are significantly different (*p* < 0.05) according to HSD tests.

## Data Availability

The datasets generated for this study are available on request to the corresponding author.

## References

[1] Kale R., Deshmukh R. (2020). 2021 Ready-to-Drink (RTD) Tea Market.

[2] Lei S., Lei S. (2020). Repurchase Behavior of College Students in Boba Tea Shops: A Review of Literature. Coll. Stud. J..

[3] Hsu W.S. (2018). The Study on the Competitive Strategies of Taiwan’s Handmade Drink Industry: The Case Study of D Group. Master’s Thesis.

[4] Jhu G.Y. (2006). Survey of the Drink and Consumption Behavior of Consumers in Taiwan. Master’s Thesis.

[5] Zeng L., Ma M., Li C., Luo L. (2017). Stability of tea polyphenols solution with different pH at different temperatures. Int. J. Food Prop..

[6] Lin S.D., Liu E.H., Mau J.L. (2008). Effect of different brewing methods on antioxidant properties of steaming green tea. LWT-Food Sci. Technol..

[7] Labbé D.P., Tremblay A., Bazinet L. (2006). Effect of Brewing Temperature and Duration on Green Tea Catechin Solubilization: Basis for production of EGC and EGCG-enriched fractions. Sep. Purif. Technol..

[8] Lin S.D., Yang J.H., Hsieh Y.J., Liu E.H., Mau J.L. (2014). Effect of different brewing methods on quality of green tea. J. Food Process. Pres..

[9] Murugesh C.S., Rastogi N.K., Subramanian R. (2018). Athermal extraction of green tea: Optimisation and kinetics of extraction of polyphenolic compounds. Innov. Food Sci. Emerg. Technol..

[10] Alencar N.M.M., Ribeiro T.G., Barone B., Barros A.P.A., Marques A.T.B., Behrens J.H. (2019). Sensory profile and check-all-that-apply (CATA) as tools for evaluating and characterizing syrah wines aged with oak chips. Food Res. Int..

[11] Castura J.C., Antunez L., Gimenez A., Ares G. (2016). Temporal check-all-that-apply (TCATA): A novel dynamic method for characterizing products. Food Qual. Prefer..

[12] Liou B.K., Jaw Y.M., Chuang G.C.C., Yau N.N.J., Zhuang Z.Y., Wang L.F. (2020). Important sensory, association, and postprandial perception attributes influencing young Taiwanese consumers’ acceptance for Taiwanese specialty teas. Foods.

[13] Dai J.R., Chiou C.S., Chen K.R., Yang M.J. (2015). The best cold brewing conditions for different types of tea. Bi-Mon. Tea News.

[14] Peryam D.R., Pilgrim F.J. (1957). Hedonic scale method of measuring food preferences. Food Technol..

[15] Pineau N., Goupil de Bouillé A., Lepage M., Lenfant F., Schlich P., Martin N. (2012). Temporal dominance of sensations: What is a good attribute list?. Food Qual. Prefer..

[16] Lee S.M., Lee H.S., Kim K.H., Kim K.O. (2009). Sensory characteristics and consumer acceptability of decaffeinated green teas. J. Food Sci..

[17] Qin Z., Pang X., Chen D., Cheng H., Hu X., Wu J. (2013). Evaluation of Chinese tea by the electronic nose and nose and gas chromatography-mass spectrometry: Correlation with sensory properties and classification according to grade level. Food Res. Int..

[18] Li H.H., Luo L.Y., Wang J., Fu D.H., Zeng L. (2019). Lexicon development and quantitative descriptive analysis of Hunan fuzhuan brick tea infusion. Food Res. Int..

[19] MacFie H.J., Bratchell N., Greenhoff K., Vallis L.V. (1989). Designs to balance the effect of order of presentation and first-order carry-over effects in Hall tests. J. Sens. Stud..

[20] Xlstat (2017). Data Analysis and Statistical Solution for Microsoft Excel.

[21] Ares G., Tárrega A., Izquierdo L., Jaeger S.R. (2014). Investigation of the number of consumers necessary to obtain stable sample and descriptor configurations from check-all-that-apply (CATA) questions. Food Qual. Prefer..

[22] Kumar R.S.S., Murugesan S., Kottur S., Gyamfi D., Preedy V.R. (2013). Black tea: The plants, processing/manufacturing and production. Tea in Health & Disease Prevention.

[23] Bhuyan L.P., Borah P., Sabhapondit S., Gogoi R., Bhattacharyya P. (2015). Spatial variability of theaflavins and thearubigins fractions and their impact on black tea quality. J. Food Sci. Technol..

[24] Obanda M., Owuora P.O., Mang’okab R., Kavoic M.M. (2004). Changes in thearubigin fractions and theaflavin levels due to variations in processing conditions and their influence on black tea liquor brightness and total colour. Food Chem..

[25] Novotny J.A., Baer D.J. (2013). Tea. Encycl. Hum. Nutr..

[26] King S.C., Meiselman H.L. (2010). Development of a method to measure consumer emotions associated with foods. Food Qual. Prefer..

[27] Bhumiratana N., Adhikari K., Chambers E. (2014). The development of an emotion lexicon for the coffee drinking experience. Food Res. Int..

[28] Wang L.F., Park S.C., Chung J.O., Baik J.H., Park S.K. (2004). The compounds contributing to the greenness of green tea. J. Food Sci..

[29] Josse J., Pagès J., Husson F. (2008). Testing the significance of the RV coefficient. Comput. Stat. Data Anal..

